# Vaccine-induced antigen archiving enhances local memory CD8+ T cell responses following an unrelated viral infection

**DOI:** 10.21203/rs.3.rs-3307809/v1

**Published:** 2023-09-25

**Authors:** Beth Tamburini, Thu Doan, Tadg Forward, Erin Lucas, Ira Fleming, Aspen Uecker-Martin, Jay Hesselberth, Thomas Morrison

**Affiliations:** University of Colorado Anschutz Medical Campus; University of Colorado Anschutz Medical Campus; University of Colorado Anschutz Medical Campus; University of Colorado Anschutz Medical Campus; University of Colorado Anschutz Medical Campus; University of Colorado Anschutz Medical Campus; University of Colorado Anschutz Medical Campus; University of Colorado

**Keywords:** lymph node, bystander activation, antigen archiving, lymph node stromal cell, protection, CD8 T cell

## Abstract

Viral and vaccine antigens persist or are archived in lymph node stromal cells (LNSC) such as lymphatic endothelial cells (LEC) and fibroblastic reticular cells (FRC). Here, we find that, during the time frame of antigen archiving, LEC apoptosis caused by a second, but unrelated, innate immune stimulus such as vaccina viral infection or CpG DNA administration boosted memory CD8+ T cells specific to the archived antigen. In contrast to ”bystander” activation associated with unrelated infections, the memory CD8+ T cells specific to the vaccine archived antigen were significantly higher than memory CD8+ T cells of a different antigen specificity. Finally, the boosted memory CD8+ T cells resulted in increased protection against *Listeria monocytogenes* expressing the vaccine antigen, but only for the duration that the vaccine antigen was archived. These findings outline a novel mechanism by which LNSC archived antigens, in addition to bystander activation, can augment memory CD8+ T cell responses during repeated inflammatory insults.

## Summary

Antigen archiving is a mechanism by which lymph node stromal cells retain antigens beyond the resolution of the immune response. A local unrelated infection results in presentation of archived antigens to memory CD8+ T cells improving protection against antigenic re-challenge.

## Introduction

Most currently available vaccines elicit neutralizing antibodies with the primary outcome of vaccine immunogenicity being assessed through surrogate markers such as antibody titers. However, antibody neutralization relies on the recognition of surface-exposed epitopes that are highly mutagenic and many pathogens can escape pre-existing antibody-mediated immunity. Specifically, rapidly mutating pathogens such as coronaviruses and influenza viruses can evade the humoral immune responses that most vaccines generate. However, the long-lasting T cell population and its diverse TCR repertoire recognize a small number of immunodominant peptides associated with numerous virus-encoded amino acid sequences that have MHC binding motifs ^1, 2, 3^. In addition to humoral responses, T cell responses are critical to induce the most efficacious protection against pathogens. In SARS-CoV-2 infections, antibodies produced during the early phase of infection decline over time ^4, 5, 6, 7, 8^ as seen in a cohort of SARS-CoV-2 convalescent patient IgG responses, which waned after 6 months, while T cell responses were stable for up to 1 year ^9, 10, 11^. Thus, T cells produce durable protective responses following vaccination resulting in viral clearance of SARS-CoV-2 ^12, 13^. Furthermore, mRNA-lipid nanoparticles (LNP) vaccines elicit both antibody and T cell-mediated responses that work synergistically to provide immunity against SARS-CoV-2 and impede disease progression ^14, 15^. Therefore, understanding factors that influence how T cell-mediated immunity is generated and re-called is critical to improving current vaccine regimens.

Many studies have established that viral-derived antigens persist for extended periods of time within lymph nodes (LN)s following viral infection ^16, 17, 18, 19, 20^. These findings have important implications for the development of vaccines and immunotherapies as they suggest that encouraging antigen persistence may be an effective strategy for boosting immune responses to viral infection. For example, influenza virus antigens that persist can recruit memory T cells, and provide protection against reinfection^16, 17, 18, 20, 21, 22^, which suggests that persisting antigens play a critical role in augmenting memory T cell responses to viral infections. Recently, we demonstrated that a subunit vaccine formulation consisting of either a TLR agonist (polyI:C) with an agonistic anti-CD40 antibody or a conjugated TLR-antigen causes the persistence of the vaccine-derived antigen in the draining LN ^23, 24, 25^. Similar to virus-derived antigen, persisting antigen from vaccines also improves T cell memory ^23^. This type of antigen persistence, which we termed “antigen archiving” ^23^, is mediated by lymph node stromal cells (LNSC)s and differs from chronic viral infections seen in patients with human immunodeficiency virus (HIV) or mouse models of lymphocytic choriomeningitis virus (LCMV) where lack of resolution of the infection leads to T cell exhaustion and eventual immune dysfunction due to chronic engagement of the adaptive immune response ^26, 27, 28, 29^.

LNSCs are comprised of three main subsets, which include fibroblastic reticular cells (FRC), lymphatic endothelial cells (LEC), and blood endothelial cells (BEC) - each of which can be subsetted further based on transcriptional profiling ^25, 30, 31, 32, 33, 34^. Different LNSCs are capable of retaining antigens. Follicular dendritic cells (FDC), a fibroblast subset, acquire antibody:antigen immune complexes (ICs), which are held in non-degradative endosomal compartments that can be recycled to the surface for antigen sampling by B cells ^35^. FDCs hold multiple different types of ICs in their recycling endosomes, allowing for a diverse range of antigens to be presented to B cells ^35, 36, 37^, resulting in the generation of robust plasma cell responses and high levels of specific antibodies that can neutralize antigens ^36^. Our previous work demonstrated that LECs have the capacity to store vaccine and viral-associated antigens for prolonged periods of time within the lymph node and that antigens retained by LECs are important for T cell protective immunity ^23, 24, 25^. By labeling antigen with nucleic acid or fluorescent tags prior to immunization, we detected antigen in the lymph node by single-cell RNA sequencing and flow cytometry predominantly in LEC subsets including floor, ceiling, collecting, and Ptx3 LECs at 2–5 weeks post-vaccination ^23, 25^. Although LEC presentation of self-antigens ^38, 39^ or non-adjuvanted antigens ^40^ is tolerogenic, we demonstrated that archived antigens are not presented by LECs directly to CD8 + T cells, but rather are transferred from LECs to migratory conventional DCs (cDCs) ^23, 24^. The specific mechanism by which antigen exchange occurs between LECs and DCs is unclear but some potential mechanisms we identified include cell-cell interactions between migratory DCs and antigen-bearing LECs, endocytosis of apoptotic LECs by the DCs ^24^, or possibly through capture of exosomes secreted by the LECs. Upon acquisition of antigens from LECs, migratory cDCs process and present antigenic peptides by MHC class I to memory CD8 + T cells ^24^. Adoptive transfer studies indicate that presentation of archived antigen to memory CD8 + T cells even at late time points after vaccination increases the number of antigen-specific memory CD8 + T cells with enhanced cytotoxic capabilities during an antigenic re-challenge with *Listeria monocytogenes (LM)*-expressing ovalbumin (ova) ^23^. Consequently, mice challenged with LM-ova had a lower bacterial burden and thus enhanced protection against infection ^23^. Thus, LEC antigen archiving is an important process by which DCs acquire foreign antigens at late time points post-vaccination or viral infection to enhance protective immunity.

While antigen archiving appeared to improve protective memory responses through the slow release of antigens during LN contraction ^24^ it was still unclear if archived antigens could be released during another inflammatory event that resulted in LEC expansion and contraction. Indeed, others have demonstrated that memory CD8 + T cells can be stimulated as a result of heterologous immunity ^1^. At least one of the mechanisms by which heterologous immunity is conferred is through ”bystander” activation. Bystander activation occurs as a result of cytokine (e.g. IFNa, IL18, IL15) produced during viral infection, but independent of antigen recognition by the T cell receptor ^41^. Bystander activation can lead to improved protection against heterologous challenge via increased production of IFNg by non-specific T cells ^42, 43^. Based on the capacity of memory CD8 + T cells to respond more readily than naïve CD8 + to lower levels of antigen and cytokines in the microenvironment ^41, 44, 45^, this phenomenon is unsurprisingly driven by memory T cells. Whether archived antigen release is an additional mechanism by which memory CD8 + T cells can be stimulated more specifically to push them into a secondary or tertiary memory state with an increased capacity to proliferate and produce cytokines ^46, 47^ are unknown.

Unresolved questions regarding the prior work include whether antigens can be released to encourage T cell responses, if an unrelated inflammatory stimulus can promote increased antigen-specific memory T cell protective responses as a result of archived antigen, and whether the benefits of antigen archiving are local or systemic. Here, we explore how LEC handling of archived antigens during an unrelated infection impacts the vaccine-induced memory T cell responses and protection. To this end, we confirmed that an unrelated innate immune stimulus such as vaccinia virus-Western Reserve (VV-WR) infection or CpG DNA stimulation caused both LEC proliferation (3–6 days) and apoptosis (2–3 weeks) ^23, 24, 48, 49^ when administered 2 weeks after a vaccination that induces antigen archiving. We found that once the vaccine antigens were archived, a secondary VV-WR infection or CpG DNA injection caused a significant increase in vaccine antigen-specific memory CD8 + T cells during the time frame of LEC apoptosis. This observed increase in antigen-specific CD8 + T cells was partly due to cytokine-induced “bystander activation” caused by the VV-WR infection. However, a further increase in vaccine antigen-specific CD8 + T cells was independent of “bystander activation” and rather a result of T cell receptor (TCR) engagement. Furthermore, the increase in CD8 + T cells was a result of archived antigen as the protective benefit caused by VV-WR during antigenic rechallenge was eliminated if VV-WR was administered beyond the time frame of antigen archiving. Interestingly, enhanced protection to the vaccine antigen, resulting from a later VV-WR infection, was only observed locally. Taken together, our data demonstrate that LEC-archived antigens have implications on downstream memory CD8 + T cell responses during an unrelated infection and identify a mechanism that leads to superior CD8 + T cell effector function during an antigenic rechallenge.

## Results

### Lymphatic endothelial cells archive antigens following vaccination.

**Figure 1:**
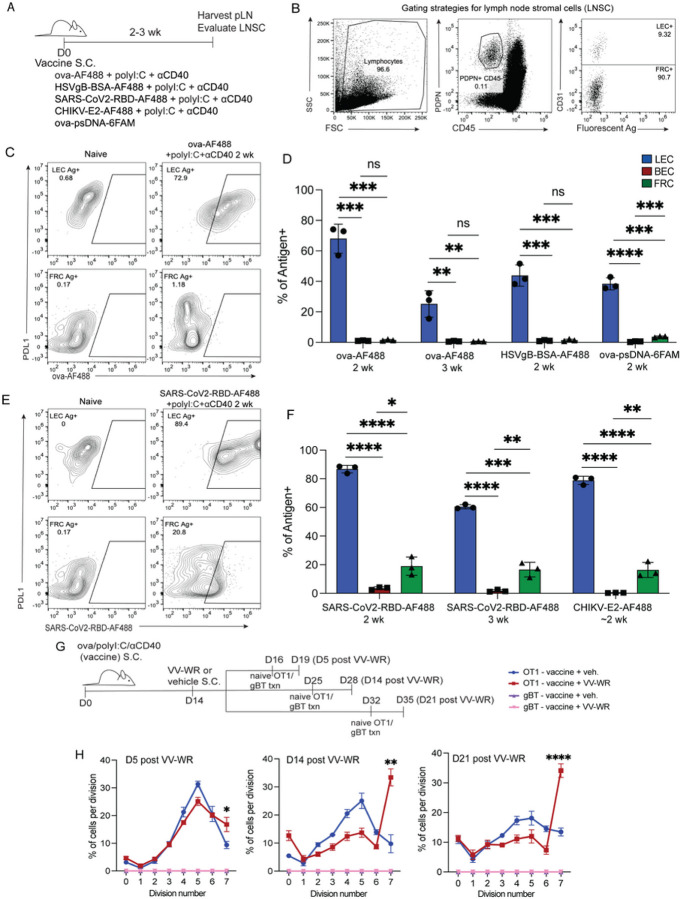
Lymphatic endothelial cells archive antigens following vaccination. (A) Experimental schematic for B-E. C57/BI6 mice were vaccinated subcutaneously in the footpad and/or flank with the indicated antigens and adjuvants. (B) Cells were stained with CD45, PDPN, CD31 and PD-L1. Gating strategies for lymph node stromal cells (LNSC). Cells were gated on CD45-PDPN+. To differentiate LEC and FRC cells were gated on CD31. Shown are LEC and FRC antigen-positive cells based on PD-L1 expression (floor, MARCO LEC). Blood endothelial cells (BEC) were gated as CD45-CD31+PDPN- (gating of BEC can be found in supplemental Figure 1). The amount of fluorescent antigen for different LNSC was determined in D and F. (C) Representative flow cytometric plots of ova-AF488+ LEC and FRC in mice 2–3 weeks after immunization with ova conjugated to Alexa-Fluor 488 (AF488) and polyI:C and aCD40 as in A. (D) Quantification of the frequency of LEC, BEC, and FRC that are positive for the indicated antigens in the popliteal LN (pLN). (E) Same as C, except for mice immunized SARS-CoV2-RBD-AF488, polyI:C, and aCD40. (F) Same as in D, except for SARS-CoV2-RBD and CHIKV-E2. CHIKV-E2 was repeated for 9–14 days post-vaccine (~2 weeks). (G) Experimental schematic for H. (H) Mice were immunized with the subunit vaccine containing ova, polyI:C, and aCD40. Two weeks later, mice were infected with vaccinia virus (VV-WR) or vehicle (PBS). Three days pre-harvest, OT1 T cells labeled with VPD or gBT T cells labeled with CFSE were transferred into previously immunized mice. The percentage of transferred OT1 or gBT T cells in each division was quantified for each specific time point for each mouse. Statistical analysis was done using an unpaired t-test where the p-value between naïve and indicated antigen is <0.0001. In each experiment, at least n=2–3 mice per group were evaluated and the experiment was repeated n=2–5 times for C-E and repeated n=2 times for G. Shown is the representative data from one of the experiments.

We previously discovered that LECs store soluble ovalbumin (ova) antigen both at the single-cell level and within whole lymph node (LN) tissue by using conjugated DNA tags as well as fluorescent tags that label the antigen ^20–22^. Here, we further build on these previous findings by showing that various protein antigens are archived for two to three weeks by LECs in the draining LN after subcutaneous immunization (Fig. 1A). By gating on CD45-cells we were able to discern the three main lymph node stromal cells (LNSC) populations: LECs, FRCs, and BECs based on the expression of podoplanin (PDPN) and CD31 (Fig. 1A,B and Supplemental Fig. 1A). To better visualize different LEC subsets we also stained cells with anti-PD-L1 which is expressed by floor and Marco + LECs ^34^. Using a number of different types of antigens and TLR agonists, we assessed antigen localization at 2–3 weeks post-vaccination. In the presence of a combination adjuvant that includes polyI:C, a TLR3 agonist, and an agonistic anti-CD40 antibody (αCD40), we confirm that LECs archive fluorescently labeled ova (Fig. 1C,D). Moreover, this observed phenomenon is not specific to ova protein as we also found that HSV-derived SSIEFARL peptide conjugated to bovine serum albumin (BSA) (HSV-gB-BSA-AF488) accumulates in LECs (Fig. 1D). To further address whether this observation was specifically polyI:C-dependent, we immunized mice with ova conjugated to phosphorothioated DNA (ova-psDNA), which engages TLR9, and observed comparable levels of LEC-associated ova to polyI:C (Fig. 1D). To assess whether different protein antigens also accumulate in LECs or other cell types, we evaluated the SARS-CoV-2 receptor binding domain (RBD) protein and the chikungunya virus E2 glycoprotein (CHIKV-E2) (Fig. 1E,F), both administered in combination with polyI:C and αCD40. We found that the SARS-CoV-2-RBD was also acquired and archived by LECs after immunization, but in contrast to albumin-based antigens was also acquired by FRCs to a lesser degree (Fig. 1E,F). Interestingly, within the FRC population, the RBD protein levels were maintained from 2 weeks to 3 weeks (Fig. 1E,F). Additionally, SARS-CoV-2-RBD was present in both PD-L1^hi and low^ LEC populations (Fig. 1E). Finally, when evaluating recombinant CHIKV-E2 we noticed that again, both LEC and a small frequency of FRCs acquired the E2 protein at ~ 2 weeks post-vaccination. Of note, CHIKV E2 is the required protein necessary for viral entry into LEC and FRC populations via the receptors MARCO ^31^ and MXRA8 ^50, 51^, respectively. Similar to CHIKV infection there was more detectable E2 within the LEC than FRC populations ^31^. There was minimal detection of antigens in BECs (Fig. 1D,F). To confirm antigen was functionally archived, we utilized TCR transgenic T cells specific for ova or HSV-gB-BSA. Ova is presented to OT1 TCR transgenic T cells, recognizing the dominant ova epitope – SIINFEKL, while the BSA-SSIEFARL is presented to gBT, recognizing the SSIEFARL epitope ^52, 53^. We transferred carboxyfluorescein succinimidyl ester (CFSE)- or violet proliferation dye (VPD)-labeled TCR transgenic T cells into mice at two to three weeks post-vaccination (Supplemental Fig. 1B–E). Three days after T cell transfer, T cell proliferation in the draining LN was assessed by CFSE or VPD dilution (Supplemental Fig. 1B–E). Both OT1 and gBT T cells responded to their cognate antigen, demonstrating the presence of archived antigens within the host 2–3 weeks post-vaccination. These data confirm that LNSCs archive a wide array of antigens during an active immune response and that there may be some cell type specificity based on the type of antigen delivered.

As we were interested in how LECs impact the downstream immune response, and based on our findings that ova is archived specifically by LECs, all remaining studies were performed with ova as the archived antigen. In response to VV-WR infection, we showed that LECs undergo apoptosis during the contraction phase of LN remodeling (Supplemental Fig. 2). Our previous studies demonstrated that LEC apoptosis is one mechanism by which archived antigens can be acquired by migratory cDCs^24^. One of the determinants of fully elicited CD8 + T cell responses is the cross-presentation of exogenous antigen on MHC Class I by conventional cDCs. We evaluated whether VV-WR infection after subunit immunization of ova would activate ova-specific memory CD8 + T cells as a response to the release of archived antigens by the LECs^24^. To evaluate this we first vaccinated mice with ova/polyI:C/aCD40 subcutaneously in the footpads, and 14 days later we infected mice in the same location with VV-WR, a strain of vaccinia virus that contains no ova-derived epitopes (Fig. 1G). We next asked if we could detect archived antigen by assessing naïve OT1 or gBT proliferation and saw that OT1 (ova-specific) CD8 + T cells divided three days post-VV-WR infection, but gBT (non-ova-specific) CD8 + T cells did not divide (Fig. 1G,H). We found that at 14 and 21 days post-VV-WR infection, there was an accumulation of OT1 T cells in the final division of VV-WR-infected mice compared to mice that did not receive VV-WR (Fig. 1H). This indicated two things, first, that there were archived antigens (ova) in the LN that were presented to naïve OT1 T cells and not gBT T cells, and second, that VV-WR infection caused the responding T cells to accumulate rather than be deleted, following division.

### Endogenous antigen-specific memory CD8 + T cells accumulate following vaccinia infection.

**Figure 2:**
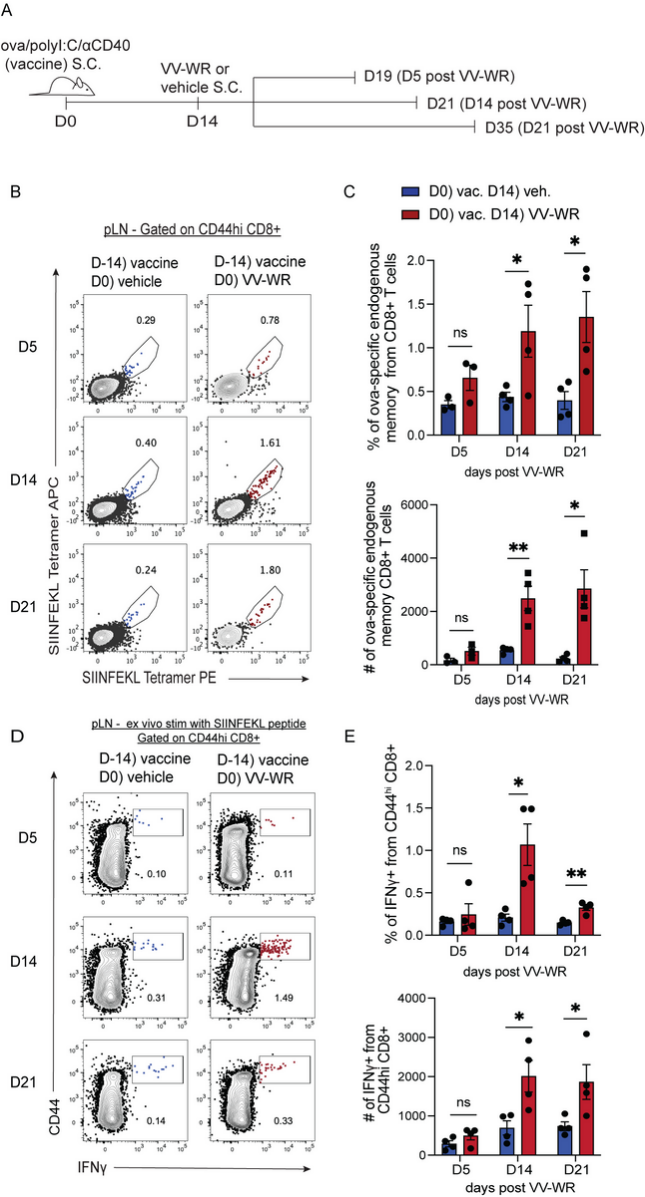
Endogenous antigen-specific memory CD8+ T cells accumulate following vaccinia infection. (A) Experimental schematic for B-E. Mice were immunized subcutaneously in the footpad with a subunit vaccine containing ova, polyI:C, and aCD40. Two weeks later, mice were infected with VV-WR or vehicle (PBS). Popliteal LNs (pLN) were harvested at respective time points post-VV-WR infection. Half the cells were used to evaluate endogenous CD8+ T cells and the other half were used for *ex vivo* stimulation with SIINFEKL peptide. (B) Representative flow cytometric plots of endogenous ova-specific H2-Kb SIINFEKL tetramer+ CD8+ T cells were evaluated using SIINFEKL-tetramer PE and SIINFEKL-tetramer APC. Prior to tetramer, cells were gated as B220-/CD8+/CD44^hi^. Blue represents mice that were injected subcutaneously with vehicle at D14 and red represents mice that were infected with VV-WR at D14. (C) Quantification of frequency and number of ova-specific endogenous memory CD8+ T cells in the draining popliteal LN. (D) Cells at respective time points were stimulated *ex vivo* with SIINFEKL peptide for 4–6 hrs to evaluate cytokine production. Respective flow cytometric plots show IFNg production of B220-/CD8+/CD44^hi^ cells. (E) Quantification of frequency and number of IFNg -producing B220-CD44^hi^ CD8+T cells from the draining pLN. Statistical analysis was done using an unpaired t-test where the p-value between vaccine + vehicle (blue bar) and vaccine + VV-WR (red bar) is <0.0001. Errors bars are mean ± standard error of the mean. In each experiment, n=3–4 mice per group were evaluated and the experiment was repeated n=3 times. Shown is the representative data from one of the experiments.

Our findings displayed in Fig. 1 suggested that VV-WR infection following immunization caused the release of antigen by LEC and resulted in the persistence of transferred naïve T cells that specifically recognized the previously archived antigen. We next asked if antigen release from LECs following an unrelated viral infection during the time frame of antigen archiving impacted the phenotype and/or function of memory CD8 + T cells *in vivo*. To answer this question, mice were vaccinated with a subunit vaccine containing ova, polyI:C, and αCD40 to establish archiving of ova. Fourteen days later, mice were infected with VV-WR to evaluate the frequency and function of ova-specific CD8 + T cells at 5, 14, or 21 days post-VV-WR infection (Fig. 2A). As these time points reflect the phases of LEC and LN expansion and contraction post-VV-WR infection as well as the amount of VV in the LN (Supplemental Fig. 2), we could further establish a time frame by which ova-specific CD8 + T cells expanded and responded to an unrelated infection through the elaboration of effector cytokines. At 5 days post-VV-WR infection, there was no significant increase in the number of ova-specific CD8 + T cells within the draining popliteal LN compared to mice that were injected with the vehicle control (Fig. 2B, C, Supplemental 3-gating). However, at 14 and 21 days post-VV-WR infection, endogenous ova-specific CD8 + T cells accumulated within the draining LN at a significantly higher degree compared to vehicle-injected mice (Fig. 2B, C). Moreover, these T cells were functionally enhanced in their ability to produce IFNg after *ex vivo* stimulation with SIINFEKL peptide (an ova-derived epitope) (Fig. 2D,E, Supplemental 3-gating). We found that the IFNg response by these ova-specific CD8 + T cells isolated from the VV-WR-infected mice was significantly higher than the uninfected mice even though neither group was challenged with the ova antigen after initial subunit immunization (Fig. 2E). To determine if this increased responsiveness to archived antigen by CD8 + T cells was a result of the potent pro-inflammatory environment caused by VV-WR infection we asked if a non-infectious inflammatory stimulus could produce the same result. We again immunized mice with ova/polyI:C/αCD40 and 2 weeks later administered CpG, a TLR9 agonist, as the secondary inflammatory stimulus in lieu of VV-WR (Supplemental Fig. 4). As with VV-WR infection, we found a significant increase in the number of ova-specific memory CD8 + T cells following local administration of CpG that was dependent on TLR9 (Supplemental Fig. 4). Together, these data suggest that endogenous memory antigen-specific CD8 T cells expand more during the time frame of LEC contraction following VV-WR infection or CpG DNA injection.

### Non-archived antigen-specific memory CD8 + T cells are stimulated in the absence of antigen after vaccinia infection to a lesser degree than archived antigen-specific memory CD8 + T cells.

**Figure 3:**
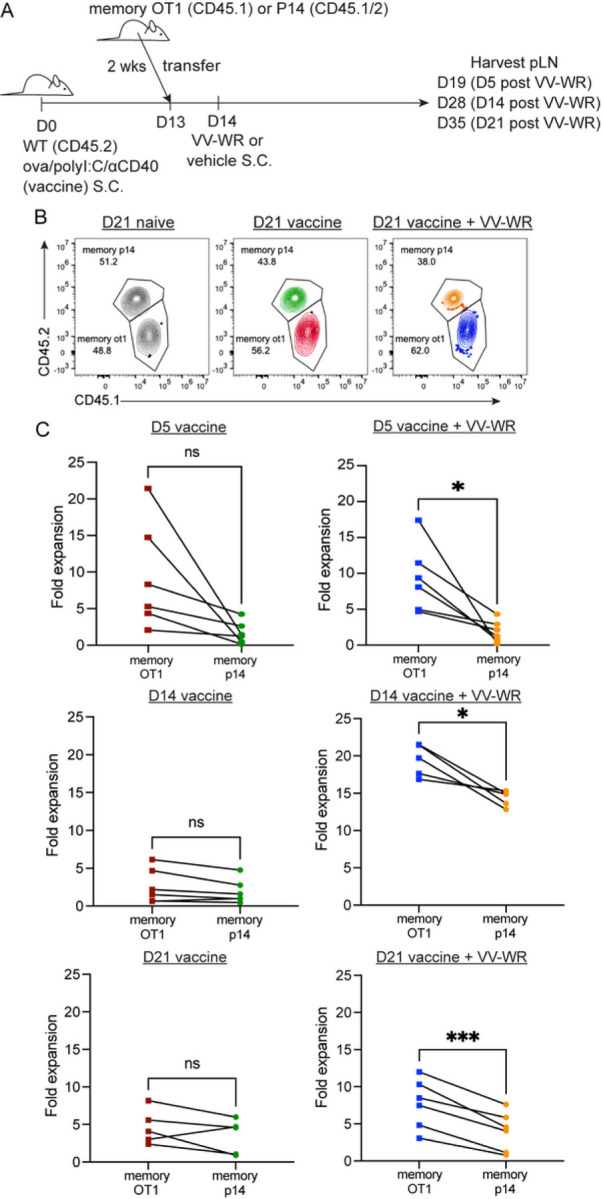
Non-archived antigen-specific memory CD8+ T cells are stimulated in the absence of antigen after vaccinia infection to a lesser degree than archived antigen-specific memory CD8+ T cells. (A) Experimental schematic for B-C. Mice were immunized and infected with VV-WR as in Figure 2. One day prior to VV-WR infection congenically different memory OT1 and memory p14 CD8+ T cells were isolated and transferred intravenously into WT mice. To establish memory, naïve OT1 or p14 cells were transferred into naïve WT mice and immunized with their cognate antigen (ovalbumin or gp33 peptide) and isolated by CD8 negative selection 2–6 weeks later as described in materials and methods. Memory OT1 and memory p14 were also transferred into naïve WT host to calculate fold expansion over OT1/p14 “take”. Popliteal LNs (pLN) were harvested and processed at indicated time points. (B) Representative flow cytometric plots of co-transferred memory p14 and memory OT1 fold expansion transferred at 1:1 ratio. (C) Memory OT1 (CD45.1/1) and p14 (CD45.1/2) were co-transferred into immunized mice (CD45.2/2) 1 day before VV-WR. The fold expansion was calculated as the total number of memory OT1 or memory p14 in antigen-bearing mice over the total number of memory OT1 or memory p14 in the naïve WT host (to accommodate for differences in ratio and “take”) at each respective time point. Statistical analysis was done using a paired t-test where the p-value between memory OT1 and memory p14 is <0.0001. In each experiment, at least n=3 mice per group were evaluated and the experiment was repeated n=2 times. In each case, a different congenic marker was used for transferred cells (e.g. OT1 was CD45.1/1 and p14 was CD45.1/2 and hosts were CD45.2/2 or OT1 was CD45.1/2 and p14 was CD45.1/1 and host was CD45.2/2). Results were similar across congenic marker combinations used. In Figure 3B, representative flow plots from one experiment are shown as an example (in the other experiment, OT1 were CD45.1/2 and p14 were CD45.1/1). Shown in Figure 3C is the combined data from both experiments. A third replicate was not performed as our experiments were adequately powered to provide statistical significance in accordance with our IACUC policies regarding animal experiments with consistent data points.

As T cells, particularly memory T cells, are able to proliferate in response to cytokine production, termed “bystander activation” ^54^ in the absence of TCR ligation, we asked if the increased T cell proliferation in Fig. 2 was a result of bystander activation. To address this, we transferred either naïve OT1 or gBT T cells into congenically distinct recipient mice 1 day prior to VV-WR infection (Supplemental Fig. 5A,B). We found that while the naïve OT1 T cells divided more at each time point after VV-WR infection compared to those that did not receive VV-WR, the gBT T cells failed to divide both with and without VV-WR infection (Supplemental Fig. 5C,D). However, because memory CD8 + T cells respond more readily than naïve CD8 T cells to both lower levels of antigen and cytokine (IL-15, IFNab^41, 44, 45^) there was a possibility that the enhanced memory CD8 + T cell activation and division were not antigen-specific but merely due to bystander activation^54^. Therefore, we asked if memory CD8 + T cells expanded as a result of antigen availability (TCR engagement) or a highly inflammatory environment due to VV-WR infection (bystander activation). To do this, mice were vaccinated with ova/polyI:C/αCD40 and infected with VV-WR two weeks later (Fig. 3A). To evaluate memory responses we generated memory OT1 or memory gp33-specific P14 T cells by transferring naïve OT1 or P14 T cells into naïve WT hosts. One day later, we immunized mice as in Fig. 3A with either gp33 peptide derived from lymphocytic choriomeningitis virus (LCMV) or ovalbumin, plus polyI:C/aCD40, and subsequently isolated these T cells. We transfered equal numbers of memory OT1 and P14 cells into naïve or ova/polyI:C/aCD40 vaccinated hosts, 2 weeks after vaccination (Fig. 3B). We chose P14 in this experiment because the TCR affinity of both OT1 and P14 T cells is high^55, 56^. In line with published findings that bystander activation occurs in the presence of infection, but not necessarily due to the presentation of cognate antigen^41, 57^, we found that the memory P14 T cells expanded as a result of VV-WR infection (Fig. 3C). When we compared the magnitude of expansion of transferred memory OT1 T cells to the expansion of the P14 T cells following VV-WR infection, we found a significant increase in the fold expansion of memory OT1 compared to memory P14 in mice at all time points (Fig. 3C). However, we only observed an increased response to VV-WR at the day 14 and day 21 time points (Fig. 3C). It appeared that the largest increase in bystander activation occurred at day 14 post-VV-WR infection. However, at day 21 we found limited T cell expansion by transferred P14 memory cells post VV-WR and a significant increase in memory OT1 cells. These data indicate that although there is an element of bystander activation attributed to VV-WR infection, particularly at 14 days post-VV-WR, bystander activation is transient and increased proliferation subsides after 21 days. These findings suggest that archived antigen presentation following VV-WR infection leads to a predominantly antigen-specific endogenous memory CD8 + T cell response. Although there are still minor levels of activated non-antigen specific T cells, we show a significantly greater expansion of memory T cells consistent with the time frame of LEC apoptosis following VV-WR infection.

### CD8 + T cells activated during vaccinia infection have increased immunogenicity following the rechallenge of previously archived antigens.

**Figure 4:**
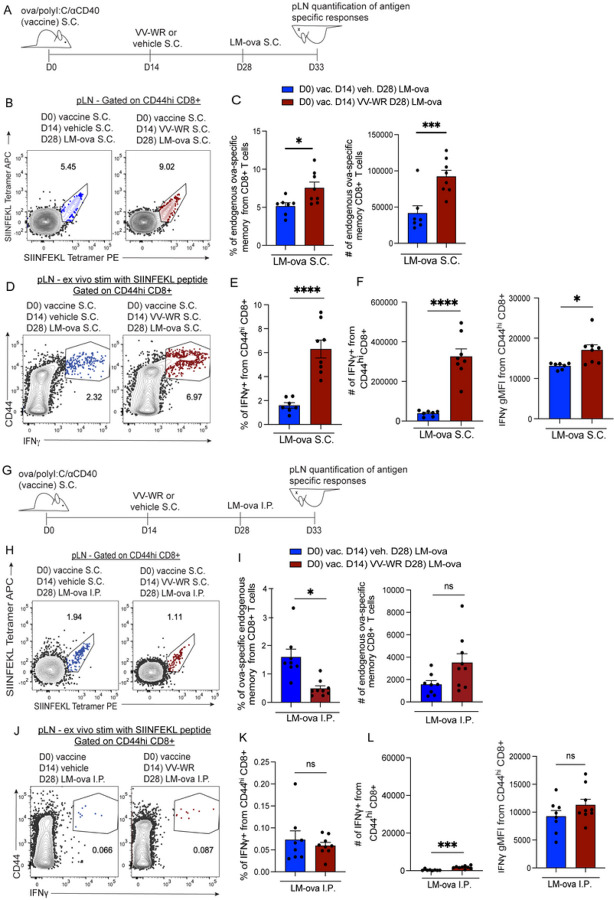
CD8+ T cells activated during vaccinia infection have increased immunogenicity following re-challenge of previously archived antigen. (A) Experimental schematic for B-F. Mice were immunized with ova/polyI:C/aCD40 vaccine and infected with VV-WR 2 weeks later. Two weeks after VV-WR, mice were challenged LM-ova subcutaneously (S.C.) Five days post-LM-ova, popliteal LN (pLN) were harvested to assess endogenous archived-antigen (ova)-specific memory CD8+ T cells in the draining pLN. (B) Representative flow plots of mice given LM-ova S.C. Blue represents mice that were injected subcutaneously with vehicle at D14 and red represents mice that were infected with VV-WR at D14. Cells were evaluated using SIINFEKL-tetramer PE and SIINFEKL-tetramer APC. Previous gates were B220-/CD8+/CD44^hi^ (C) Quantification of frequency and the total number of ova-specific endogenous memory CD8+ T cells in the popliteal LN. (D) Representative flow cytometric plots of mice given LM-ova S.C. The cells were stimulated *ex vivo* with SIINFEKL peptide for 4–6 hrs to evaluate cytokine production. Respective flow cytometric plots show IFNg production of cells gated previously on B220-/CD8+/CD44^hi^. (E) Quantification of frequency IFNg -producing from CD44^hi^ CD8+ T cells in the draining popliteal LN from the shown gate. (F) Quantification of the total number and geometric mean fluorescence intensity (gMFI) of IFNg -producing from CD44^hi^ CD8+T cells in the draining pLN from the shown gate. (G) Experimental schematic for H-L. Mice were challenged with LM-ova intraperitoneally (I.P.) (H) Same as B, except for the mice were challenged with LM-ova I.P. (I), Same as C, except for the mice were challenged with LM-ova I.P. (J) Same as D, except for the mice were challenged with LM-ova I.P. (K) Same as E, except for the mice were challenged with LM-ova I.P. (L), Same as F, except mice were challenged with LM-ova I.P. Statistical analysis was done using an unpaired t-test where the p-value between vaccine + vehicle + LM-ova (blue bar) and vaccine + VV-WR + LM-ova (red bar) is <0.0001. Errors bars are mean ± standard error of the mean. In each experiment, n=3–5 mice per group were evaluated and the experiment was repeated n=2 times. Shown is the combined data from both experiments. A third replicate was not performed as our experiments were adequately powered to provide statistical significance in accordance with our IACUC policies regarding animal experiments with consistent data points.

We previously identified that archived antigens enhance protective immune responses by increasing IFNg and IL-2 production during antigenic rechallenge^23^. Thus, we next asked if mice with archived antigens that received an inflammatory stimulus (VV-WR) to induce antigen release were better protected against an antigenic re-challenge. To this end, mice previously vaccinated with ova/polyI:C/aCD40, that did or did not receive VV-WR 2 weeks later, were challenged with a recombinant strain of *Listeria monocytogenes* that expresses ovalbumin (LM-ova) either locally (subcutaneously in the footpad) (Fig. 4A) or systemically (intraperitoneally) (Fig. 4G). Upon LM-ova challenge, we saw an increase in both the frequency and number of antigen-specific CD8 + T cells in the draining LN as assessed by SIINFEKL tetramer staining (Fig. 4B, C). Additionally, we found that responding CD8 + T cells had a significantly higher frequency of IFNg-producing cells (Fig. 4D,E). We also note that of the cells expressing IFNg, more IFNg was produced than their non-VV-WR infected counterparts (Fig. 4D,F). This is consistent with published data demonstrating that antigen-specific tertiary memory CD8 + T cells display increased cytokine production compared to antigen-specific primary memory CD8 + T cells ^58^. This was in contrast to the response seen during systemic infection (Fig. 4G) where there was no significant difference in the number of antigen-specific T cells in the vaccine-draining LN (Fig. 4H,I) nor in the frequency of cells producing IFNg (Fig. 4K). The number of IFNg-producing cells was higher, but strikingly low in number compared to the draining LN (Fig. 4F,L) while the amount of IFNg produced was no different following LM-ova IP challenge as indicated by mean fluorescence intensity (Fig. 4L). Similarly, there was no difference in antigen-specific cell frequency or number or IFNg production in the spleen of mice who were challenged either subcutaneously or intraperitoneally (I.P.) with LM-ova (Supplemental Fig. 6). These findings establish that vaccine antigen-specific CD8 + T cells are recalled locally during a pathogenic rechallenge following an unrelated inflammatory stimulus (VV-WR) as seen by increased numbers of responding ova-specific CD8 + T cells that possess the ability to produce high levels of IFNg (Fig. 4D,F).

### Vaccinia infection within the duration of antigen archiving induces robust and durable protective immunity.

**Figure 5:**
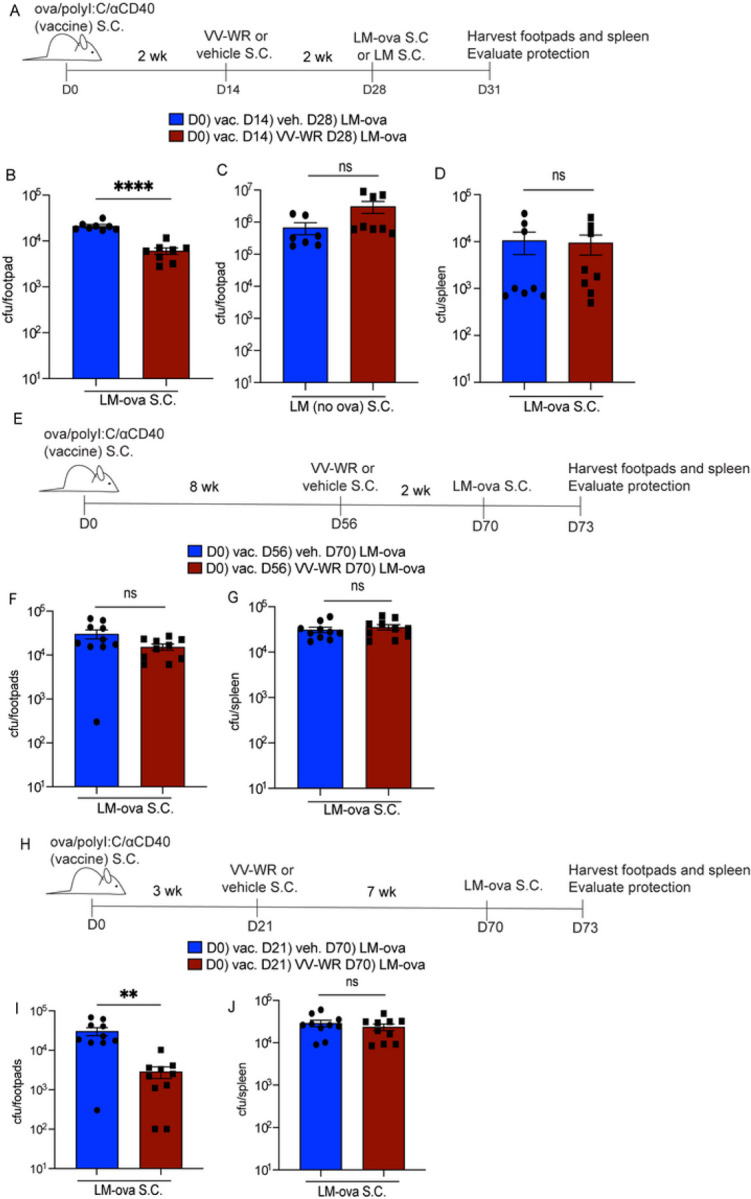
Vaccinia infection during the timeframe of antigen archiving induces robust and durable protective immunity. (A) Experimental schematic for B-D. Mice were immunized with ova/polyI:C/aCD40, infected with VV-WR, and challenged with LM-ova or LM at indicated time points. Foot and ankle skin or spleen were harvested. (B-D) Respective tissues were processed as described in the methods section. Homogenized tissues were plated on BHI + erythromycin (LM-ova) or streptomycin (LM) plates and colonies were counted after 3 days of growth. (E) Experimental schematic for F, G. Mice were immunized, infected with VV-WR, and challenged with LM-ova at indicated time points. (F, G) Same as B-D. (H) Experimental schematic for I, J. Mice were immunized, infected with VV-WR, and rechallenged with LM-ova at indicated time points. (I, J) Same as B-D. Statistical analysis was done using unpaired t-test where the p-value between vaccine + vehicle (blue bar) and vaccine + VV-WR (red bar) is <0.0001. Errors bars are mean ± standard error of the mean. In each experiment, at least n=3–5 mice per group were evaluated and the experiment was repeated n=2 times. Shown are all data points from both experiments. A third replicate was not performed as our experiments were adequately powered to provide statistical significance in accordance with our IACUC policies regarding animal experiments with consistent data points.

We next asked if the increase in the number of CD8 + T cells with enhanced effector function limited bacterial burden at the site of infection after VV-WR infection (Fig. 5A). Indeed, vaccinated mice previously infected with VV-WR and then rechallenged with LM-ova demonstrated a small, but significant and repeatable reduction in colony-forming units (CFU) of LM-ova in the skin of the footpads compared to mice that did not receive VV-WR initially (Fig. 5B). This protective phenotype was dependent on the originally archived antigen as we did not detect a significant difference in CFU from mice infected with LM that did not express ova regardless of whether they were infected with VV-WR or not two weeks prior (Fig. 5C). In parallel with the observed T cell phenotypic and functional assays assessed after systemic LM-ova infection, we found no difference in protection in the spleen of mice infected with LM-ova either subcutaneously (Fig. 5D) or intraperitoneally (Supplemental Fig. 6G). These data suggest that memory ova-specific CD8 + T cells are primed locally by the release of ova by the LECs during VV-WR infection and that the memory ova-specific CD8 + T cells increase protection against a homologous re-challenge (LM-ova), but not a heterologous re-challenge (LM-no ova).

Thus far we have shown that we can induce LEC apoptosis in order to facilitate activation of antigen-specific T cells to accumulate with enhanced effector cytokine responses during rechallenge to ova-expressing pathogens. We next evaluated the longevity by which these downstream memory T cell responses can occur in order to improve local protection upon encounter of a cutaneous pathogen during antigenic re-challenge by assessing how VV-WR infection influenced downstream effector CD8 + T cell responses at a time point after archived antigen is no longer detectable. To evaluate when archived antigen was no longer available for transferred OT1 T cells to respond to, mice were vaccinated with ova/polyI:C/aCD40, and 3 or 8 weeks later VPD labeled OT1 T cells were transferred into vaccinated mice. We found that while at 3 weeks post-subunit vaccination there was robust OT1 division, at 8 weeks there was no longer OT1 division (Supplemental Fig. 7). This demonstrates that T cells could only respond to the archived antigen remaining in the LN for less than 8 weeks following ova/polyI:C/aCD40. Based on the time frame during which antigen remains archived within LECs, we infected mice with VV-WR at 8 weeks post-vaccination and rechallenged the mice with LM-ova 3 weeks post-VV-WR (Fig. 5E). We saw no significant difference in bacterial burden whether or not mice were infected with VV-WR prior to the LM-ova rechallenge (Fig. 5F). There was also no significant difference in CFUs in the spleen between mice that were infected with VV-WR and non-infected mice (Fig. 5G). Thus, when local archived antigens are not available to stimulate memory CD8 + T cells during an additional inflammatory event the protective capacity against a pathogen expressing the previously archived antigen is no longer present.

However, because we saw bystander activation peaking two weeks post VV-WR it was possible that the increased protection against LM-ova 2 weeks post VV-WR (Fig. 5B) was a result of bystander activation. To test this idea, we infected mice with VV-WR at 3 weeks post-vaccination and 7 weeks later (Fig. 5H), beyond the time frame of VV-WR-induced regulation of cytokines associated with bystander activation ^41,^ 44, 45 we evaluated protection against LM-ova in the skin and distantly in the spleen. Importantly, at 7 weeks post-VV-WR infection, the virus infection has fully resolved with the resulting cytokine profile also returning to homeostatic levels ^59, 60^. We observed a significant reduction in CFU in the footpads of mice infected with VV-WR and thus better local protection compared to mice that were not infected with VV-WR (Fig. 5I). This suggests that following VV-WR infection, the memory T cells we identified in Figs. 3 and 4 are more protective against antigenic challenge at the tissue site as a result of the recognition of their cognate antigen in the draining LN. However, we further establish that this protective phenotype, mediated by memory T cells is specific to local re-challenge as there was no increase in protection in the spleen (Fig. 5J). These findings demonstrate that archived-antigen-specific (ova) T cells can be stimulated by archived ova during a secondary inflammatory insult and that these stimulated antigen-specific T cells can maintain protective responses locally during pathogenic rechallenge in a durable manner.

## Discussion

In this study, we established a model by which we can boost cell-mediated immunity through the presentation of previously archived antigens stored in LECs. We demonstrate an increased benefit in protective immunity via the stimulation of vaccine-specific CD8 + T cells during an antigenically unrelated infection or stimulus. We propose that the memory CD8 + T cells are boosted by the antigen archived within the LECs, during the inflammatory event, as a result of LEC apoptosis and DC activation. LEC apoptosis and activation of DCs during the inflammatory stimulus and LN contraction stimulate CD8 + T cells by cross-presenting to migratory cDC1s. This is evidenced by the increased amounts of IFNg produced by the expanded memory CD8 + T cells following VV-WR infection as well as the increased protection seen with a lower bacterial burden during a pathogenic re-challenge with the vaccine archived antigen. While we also show that non-antigen-specific memory T cells (P14 or gBT) expand to a degree in response to cytokine stimulation (i.e., bystander activation ^41, 45^), we go on to show that archived antigens are a more substantial modulator of CD8 + T cell memory activation (Fig. 3). Indeed, the difference in protection that we find is only at the tissue site where the vaccination was administered. These findings suggest that the memory CD8 + T cells traffic back to the site of infection to exert cytotoxic functions locally and to protect against the insult at the site of initial infection. Furthermore, in our findings, we also identify a specific time frame by which VV-WR must be administered in order for the protective benefits of memory CD8 + T cells to occur. Beyond the time frame of antigen archiving, we do not detect any appreciable differences in bacterial load, even at the local site of vaccination.

In assessing the contribution of antigen archiving to multiple sequential infections, we considered an unrelated viral infection as a potential method to increase LEC apoptosis and promote antigen release ^24^ in addition to activating DC migration. LECs have been demonstrated to expand and contract following lymph node expansion and contraction^48, 61, 62^. This is an important feature for DC and neutrophil recruitment to the LN during infection or vaccination where LECs also express the chemokine ligand CCL21^63 64^. Consistent with these findings we indeed show that LECs that have gone through the same vaccination and infection timeline do undergo increased apoptosis at the 14 and 21 day time points, and that this is independent of previous vaccination (Supplemental Fig. 2). This timing is consistent with lymph node expansion and contraction as the immune response is activated and resolved (Supplemental Fig. 2B). Whether the LECs undergo apoptosis due to a return to homeostasis or as a result of viral infection is still unknown. Regarding vaccinia infection, while the cell entry receptor for vaccinia virus is not well defined, there is evidence that the scavenger receptor MARCO contributes to viral entry into keratinocytes. Since some LEC subsets express MARCO it is possible that one mechanism of apoptosis is through vaccinia infection of the LECs. However, we have been unable to detect virus within primary murine LEC cultures. Furthermore, the specific process and mechanism by which antigen exchange occurs between LECs and DCs remains unclear. A possibility is that apoptotic LECs release extracellular vesicles^65^, and these vesicles undergo uptake by migratory DCs that encounter these apoptotic bodies within the subcapsular sinus of the LN. Additional mechanisms may include DC trogocytosis or cytoplasm exchange^66^ of archived antigens from LECs ^67^, in order to facilitate DC acquisition of archived antigens. These processes may happen to a lesser degree in the absence of infection or inflammation to maintain a long-lasting memory CD8 + T cell pool that can rapidly respond to pathogenic infection occurring within distal sites of the LN ^68^ and exert cytotoxic functions as we have shown ^23^.

Data shown here suggests that the addition of an inflammatory stimulus to the process of antigen exchange between LECs and DCs could be affecting this mechanism in a number of ways. One mechanism could be that the inflammatory stimulus may increase antigen release from LECs to further enhance acquisition and presentation by DCs. A second possibility is that the inflammatory stimulus could also increase the frequency of LEC-DC interactions as certain inflammatory stimuli increase the amounts of migratory DCs arriving in the draining LN from the site of the infection or inflammatory stimulus. A third possibility could be that the inflammatory stimuli further activate resting LN-resident DCs to provide the required cytokines and co-stimulatory molecules. This would provide the necessary signal for the responding CD8 + T cells to establish a secondary memory function i.e. increased IFNg production upon rechallenge ^46, 47^.

The divergent pathways that allow for memory CD8 + T cells to have a superior ability to control pathogens during secondary infection through increased proliferation and elaboration of effector cytokines, especially during the time frame of antigen archiving, is still unknown. However, based on these studies, it would be pertinent to evaluate which kind of adjuvant would be most beneficial in promoting T-cell mediated immunity in order to initiate the most robust and durable CD8 + T cell memory response to protect against severe disease and pathogens. A prime example of this is the observed T cell memory responses evaluated following the SARS-CoV2 mRNA lipid nanoparticle (LNP) vaccination where protection against severe disease by T cells happens in the face of waning antibody titers, which has been critical for patient survival ^69^.

As mentioned above, the process of antigen archiving by LECs and possibly by other lymph node stromal cells (such as FDCs and or other FRC subsets) appears to be most beneficial to the host during the memory phase of the immune response. As such, it is important to further characterize which types of currently available vaccines are able to induce antigen archiving and which specific properties of LECs allow them to archive antigens in a non-degraded state. To begin to fully understand which vaccine types are capable of eliciting antigen archiving, we found that all TLR agonist-adjuvanted vaccines we have tested are capable (Figs. 1 and ^23, 25^), but whether mRNA-based vaccines contained within LNPs, viral vector vaccines, virus-like particles or others result in antigen archiving is currently unknown. Many current vaccines utilize aluminum salt (alum) as an immune adjuvant, which has been successful at initiating robust antibody-dependent responses to the antigen administered with the help of CD4 + T cells, however, cell-mediated immunity through robust CD8 + T cell responses are minimal with these current vaccine strategies ^70^. It is unlikely that the antigen administered with alum is archived like subunit vaccines, but rather forms an antigen depot, perhaps unimportant for the immune response ^71^, at the injection site rather than a bolus of antigen that can be received by the LECs within the draining LN. Furthermore, as we have also published that a concomitant T cell response is required for antigen archiving, it seems unlikely that alum provides the same protective benefit due to the minimal T cell response ^23^, however, this has yet to be tested. Future studies aimed at investigating how LECs and other LNSCs are capable of archiving non-degraded antigens and maintaining them for extended periods of time are necessary. Our single-cell sequencing analysis revealed that the genes Cavin1 and Cavin2 were upregulated in antigen-positive LECs ^25^, but not in hematopoietic populations. Previous literature has established that caveolin-mediated endocytosis depends on Caveolin1 (CAV1) and Caveolin2 (CAV2) at the membrane, which interact with Cavin1 (CVN1) and Cavin2 (CVN2) to stabilize caveolae^72^. These findings support a possible model where LECs retain antigen in non-degradative endosomes over long periods of time ^73^, unlike DCs, because caveolin-mediated endocytosis differs from pinocytosis, macropinocytosis, receptor-mediated endocytosis, and phagocytosis, in that caveosomes are specially equipped to retain endocytosed proteins. Caveosomes maintain a neutral pH, with cargo able to remain in caveosomes until either transcytosis/recycling or lysosomal degradation via RAB5-dependent fusion with the early endosome ^74^. Indeed, we found that blocking caveolin-mediated endocytosis with nystatin led to a significant decrease in antigen acquisition by LECs *in vivo*^25^. Our findings using single-cell mRNA sequencing analysis revealed caveolin-mediated endocytosis proteins to be upregulated at both early and late time points during the timeframe in which LECs are antigen-positive ^25^. We are currently investigating whether we can skew LECs toward caveolin-mediated endocytosis as a means to prolong antigen archiving and thus achieve a more durable and lasting memory T cell response that we observe within this study upon reinfection. These studies inform vaccine design, specifically geared at improving memory CD8 + T cell response to vaccination.

Collectively, here and in our prior work we have demonstrated that antigen archiving provides a unique purpose in enhancing memory CD8 + T cell function, particularly when an unrelated inflammatory stimulus is involved in order to further enhance and drive memory T cell response upon reinfection at a distal site, such as the skin. While we do not claim that antigen archiving is required for memory formation or maintenance, as was previously demonstrated ^75^. We provide a novel purpose for antigen archiving in enhancing T cell-mediated immunity and exemplify how non-canonical immune cells, like LECs, contribute to vaccine-elicited immunity and encourage protection against antigenically related pathogens. We speculate that these findings may have application to vaccinations in patients who are infected by an unrelated pathogen during the course of vaccine antigen archiving. This should be an additional factor to consider when determining optimal immunization platforms and routes because the effect of antigen archiving is specifically local this may also be pertinent for inhaled vaccines. Together, the findings outlined in this manuscript are important to consider when evaluating immune memory, particularly CD8 + T cell memory following vaccination or viral infection, especially the contribution of LNSC to immunity, and potential avenues that could be considered to employ LNSC or LEC functions to improve vaccine-mediated immunity.

## Methods

### Mice

All animal procedures were approved by the Institutional Animal Care and Use Committee at the University of Colorado Anschutz Medical Campus. 5–8 week-old male or female mice were purchased from Charles River or Jackson Labs and used at ages between 6 and 10 weeks and were bred and housed in the University of Colorado Anschutz Medical Campus Animal Barrier Facility. Wild type, OT1, P14, and gBT mice were all bred on a C57BL/6 background. OT1 mice are a TCR transgenic strain specific to the SIINFEKL peptide of ova (OVA257–264) in the context of H-2K^b^. P14 mice are a TCR transgenic strain specific to the gp33 peptide. gBT-1 (gBT) mice are a TCR transgenic strain specific to the SSIEFARL peptide of herpes simplex virus glycoprotein B (HSV-1 gB 498–505) in the context of H-2K^b^. No differences in sex or age were found in experiments.

### Vaccines and pathogen challenge

Mice were immunized subcutaneously in each footpad with the indicated protein antigen (amount administered in parenthesis), 5 µg polyI:C, and 5 µg αCD40. Ova (10µg) was purchased from Sigma-Aldrich (Cat No. A5503) and Chikungunya virus envelope 2 protein (8µg) (CHIKV-E2, strain SL-CK1) was purchased from Sino Biological (Cat. No. 40440-V08B). HSVgB-BSA (10µg) was made by combining 10 mg of maleimide-activated bovine serum albumin (BSA) (Thermo Fisher Cat. No. 77115) with 15 mg of SSIEFARL gBT peptide for 2 hours at room temperature. The conjugated HSVgB-BSA was enriched and concentrated using a 30kDa size exclusion column. SARS-CoV2-RBD protein was generated by the University of Colorado Cell Technologies Shared Resource Core. SARS-CoV2-RBD (8µg) (GenBank: MT380724.1) was made by transfecting HEK293 T cells with a His-tagged vector and the protein was purified over ATKA nickel column. For immunization with fluorescent antigens, ova, HSVgB-BSA, SARS-CoV2-RBD, and CHIKV-E2 were conjugated to AlexaFluor-488 via NHS Ester kit (Thermo Fisher Cat. No. A20000). Ova-psDNA (10µg) was created as previously described ^25^ with the addition of a fluorescein molecule conjugated to the psDNA (ova-psDNA-6FAM) for visualization using flow cytometry. Endotoxin levels were determined using the amebocyte lysate method using a Pierce^™^ Chromogenic Endotoxin Quant Kit (Thermo Scientific, Cat. No. A39553) to be less than 1 endotoxin unit (EU) per milligram of protein. When necessary endotoxin removal was performed using the protocol from Aida & Pabst^76^. Briefly, 40mg/mL of protein in PBS was incubated with 1% Triton X-114 (Sigma Aldrich, Cat. No. X114) on ice for 5 minutes, then 37°C for 5 minutes. The mixture was spun at 2095 xg with no brake for 5 minutes at room temperature, and the top layer was collected. This process was repeated for a total of three times. To remove excess Triton from the endotoxin-depleted protein, the depleted protein was incubated with hydrophobic Bio-Beads SM-2 Adsorbents (Bio-Rad, Cat. No. 1523920) overnight at 4°C. For the viral challenge, mice were infected with 10^4^ plaque-forming units (pfu) per footpad of Vaccinia Virus Western Reserve strain. For subcutaneous re-challenge with *Listeria monocytogenes* (LM) or LM expressing ova (LM-ova), the bacteria were grown in Brain Heart Infusion media from a frozen stock overnight with streptomycin (LM) or erythromycin (LM-ova) and sub-cultured for 1–4 h until the bacterial culture reached an optical density (OD) at 600 nm wavelength of 0.3–0.5. Calculating 1E9 per 1.0 OD, mice were injected with 5e5 per footpad in 50µl.

### Tetramer and intracellular cytokine staining

Draining LNs and spleens were harvested and processed by frosted glass slide maceration. Red blood cells from the spleens were lysed using Ammonium-Chloride-Potassium (ACK) lysis buffer. The cells were filtered, washed, and suspended in complete RPMI with 2.5% fetal bovine serum (FBS). Cells were stained with anti-mouse CD8 antibody (clone: 53 − 6.7) and both SIINFEKL tetramer-PE and SIINFEKL tetramer-APC (NIH tetramer core facility) for 1 h at 37°C. Cells were then stained for additional surface markers (CD44, B220, KLRG1, CD127-see table for clone numbers) for 30 min at 37°C. After washing, samples were run on BD Canto II flow cytometer or Beckman Coulter Cytoflex LX flow cytometer. For intracellular cytokine staining, single-cell suspensions were ex vivo stimulated in brefeldin A (1 µg/ml)) with or without (2 µg/ml) SIINFEKL peptide for 4–6 h at 37°C. After stimulation, cells were stained with anti-CD8, -B220, -CD3, and -CD44 antibodies (see table). Cells were then fixed with 1% paraformaldehyde and 3% sucrose for 10 min in the dark at room temperature. Cells were washed twice with FACS buffer (0.1% bovine serum albumin (BSA), 1x Hank’s buffered saline solution, 2 mM ethylene diamine tetra acetic acid (EDTA) and 0.02% sodium azide) and then permeabilized with 1x perm wash (BD Cat. No. 554723). The cells were then stained for IFNg (clone: XMG1.2) in 1x perm wash. The following day, the cells were washed in perm buffer 2 times and resuspended in FACS buffer before acquiring by flow cytometry. All flow cytometry data were analyzed with FlowJo software and statistical analysis and graphing was done using Graphpad Prism software. See the list of antibodies used in the table for reference.

### Stromal cells harvesting and staining

Draining LNs were harvested into Click’s EHAA media (FUJIfilm) and minced with 22-gauge needles. Tissues were digested in 0.25 mg of liberase dispase low (DL) (Sigma-Aldrich Cat. No. 5466202001) and 17 µg/ml DNAse (Worthington Biochemical Cat. No. LS002145) for 1 h at 37°C with pipetting every 15 min to physically agitate the digested tissues. Following digestion, cells were filtered through a 100-micron screen and washed with 5 mM EDTA and 2.5% FBS in EHAA media to stop the digestion. Cells were washed once with PBS before staining in live/dead GhostRed stain for 30 min at 4°C. Cells were then washed with FACS buffer and stained with anti-mouse CD45, CD31, and podoplanin and PD-L1 antibodies in 10% 24G2 (Fc Block) for 30 min at 4°C. Cells were washed twice with FACS buffer and run on BD Canto II flow cytometer or Beckman Coulter Cytoflex LX flow cytometer.

### Protection assay

Footpads and spleens were harvested in 2.5% NP-40 in PBS. The spleen was mascerated mechanically by grinding between two frosted glass slides. The skin of each footpad was removed from the bones and homogenized with a tissue homogenizer. Ground tissues were diluted 1:10, 1:1000, 1:10000 with PBS. All dilutions were either plated onto Bacto-Brain Heart Infusion (BHI) plates with 5 ug/mL erythromycin for LM-ova selection or plated on BHI with 50 ug/mL streptomycin for LM selection. Plates were incubated at 37°C for 1–3 days and colonies were counted.

### OT1, gBT, and P14 isolation and transfer

OT1, gBT, and P14 CD8 + T cells were isolated using the Mojosort CD8 T cell isolation kit (Biolegend Cat. No. 480008). After CD8 negative selection, the cells were labeled with VPD or CFSE to assess proliferation. For generating memory OT1, memory P14, or memory gBT, naïve T cells were isolated as described above, 1e5 cells were intravenously transferred into WT mice of a different congenic background and the following day the immunized mice were intravenously injected with the following to expand each respective transgenic T cells: 100 µg ova, 50 µg polyI:C, and 50 µg anti-CD40 for memory OT1; 100 µg HSVgb peptide, SSIERFARL, 50 µg polyI:C, and 50 µg anti-CD40 for memory gBT; 100 µg gp33 peptide, 50 µg polyI:C, and 50 µg anti-CD40 for memory P14. After 2–4 weeks, generated memory CD8 + T cells were isolated from the mice and isolated by negative selection using Mojosort CD8 T cell isolation. Antigen specific CD8 + cell frequency and number was quantified with respective tetramers (NIH core tetramer facility) by flow cytometry and ~ 8E5–1E6 cells were transferred at a 1:1 ratio into immunized mice as described in Fig. 4A.

### Statistical analysis

Statistical analysis was done using an unpaired Student’s t-test, paired Student’s t-test and two-way ANOVA in Graphpad Prism 9. *p*-values are denoted in the figure legends and in the figure images, where one asterisk represents a *p*-value of < 0.05 and two asterisks a *p*-value of < 0.01, and three asterisks a *p*-value of < 0.001. Each analysis was done with at least three mice per treatment group and each experiment was done at least twice with the same results. Error bars are mean ± the standard error of the mean.

## Supplementary Material

Supplement 1

## Figures and Tables

**Table:** Antibodies/Reagents

Reagent Type	Designation	Source or reference	Clone	Additional information
Chemical compound	Violet proliferation dye	BD Biosciences	-	-
Chemical compound	CFSE	BD Biosciences	-	-
Chemical compound	PolyI:C	Invivogen	-	for subcutaneous injections, use 5 µg/mouse; for intraperitoneal injections, use 50 µg /mouse
Antibody	Anti-mouse CD40 (Rat monoclonal)	BioXcell	FGK4.5	for subcutaneous injections, use 5 µg/mouse; for intraperitoneal injections, use 50 µg/mouse
Antibody	Anti-mouse CD8 APC-cy7 (Rat monoclonal)	Biolegend	53 − 6.7	Dilution – 1:300
Antibody	Anti-mouse CD8 BV785 (Rat monoclonal)	Biolegend	53 − 6.7	Dilution − 1:200
Antibody	Anti-mouse/human B220/CD45R BV510 (Rat monoclonal)	Biolegend	RA3–6B2	Dilution − 1:200
Antibody	Anti-mouse CD3 BV510 (Rat monoclonal)	Biolegend	17A2	Dilution − 1:200
Antibody	Anti-mouse CD44 PacBlue (Rat monoclonal)	Biolegend	IM7	Dilution − 1:200
Antibody	Anti-mouse CD44 PerCP-Cy5.5 (Rat monoclonal)	Biolegend	IM7	Dilution − 1:400
Antibody	IFNg APC	Biolegend	XMG1.2	Dilution − 1:200
Antibody	Vb5 Pe-cy7	Biolegend	MR9–4	Dilution − 1:200
Antibody	Vb8 FITC	Biolegend	KJ16–133.18	Dilution − 1:200
Antibody	Anti-mouse CD45.1 PerCP-cy5.5 (Mouse monoclonal)	Biolegend	A-20	Dilution − 1:300
Antibody	Anti-mouse CD45.2 PacBlue (Mouse monoclonal)	Biolegend	104	Dilution − 1:200
Antibody	Anti-mouse CD45 APC-cy7 (Rat monoclonal)	Biolegend	30-F11	Dilution − 1:300
Antibody	Anti-mouse CD31 PerCP-cy5.5	Biolegend	390	Dilution − 1:200
Antibody	Anti-mouse 1 BV421 (Rat monoclonal)	Biolegend	10F.9G2	Dilution – 1:200
Antibody	Anti-mouse podoplanin/gp38 APC	Biolegend	8.1.1	Dilution − 1:200
Mouse strain, background (*Mus musculus*)	WT – C57BL/6	Charles River Labs or Jackson Labs	-	-
Mouse strain, background (*Mus musculus*)	OT1 - C57BL/6-Tg(TcraTcrb) 1100Mjb/J	Jackson Labs	-	-
Mouse strain, background (*Mus musculus*)	P14	Kind gift from Raul Torres, Univeristy of Colorado Anschutz	-	-
Mouse strain, background (*Mus musculus*)	gBT-1 (gBT)	Kind gift from Bill Heath, University of Melbourne	-	-
