# Supplementary material for: Vaccine-induced antigen archiving enhances local memory CD8+ T cell responses following an unrelated viral infection
Source: Res Sq. 2023 Sep 25:rs.3.rs-3307809. Preprint. [Version 1] doi: 10.21203/rs.3.rs-3307809/v1 (PMC10571600; doi:10.21203/rs.3.rs-3307809/v1)
Supplement: Supplement 1 [file NIHPPrs3307809v1-supplement-1.pdf]

Supplemental Material and Methods

In Supplemental Fig. 2, tissues were harvested and processed as “Stromal Cells Harvesting and Staining” in the Methods section. Cells were washed once with PBS before staining in live/dead GhostRed stain (Tonbo Bioscences Cat. No. 50-105-2988) for 30 min at 4°C. Cells were then washed with FACS buffer and stained with CD45, CD31, and podoplanin and PD-L1 anti-mouse antibodies in 10% 24G2 (Fc Block) for 30 min at 4°C. Cells were washed with FACS buffer and stained with CellEvent Caspase-3/7 Green Flow Cytometry Assay kit (Thermo Fisher Cat. No. C10427) for 25 min at 37°C. Cells were run onto the cytometer without washing. For the viral plaque assay in Supplemental Fig. 2, 1-1.5e6 vero cells per well were seeded in 24-well plate in 0.5 mL of complete MEM with 5% FBS overnight. Popliteal lymph node were harvested and homogenized with the tissues grinder. Mix homogenized tissues with 0.25% trypsin at 1:1 ratio and incubate for 37°C for 1 hr. Dilute the mixture at 1:1000 with PBS. Add 50 uL of neat or

1:1000 homogenized tissues + trypsin mixture to each well in triplicate. Incubate for 2 days at 37°C. Remove media and add 0.5 mL of 10% buffered formalin and incubate for 5 min at room temperature. Aspirate formalin and add 0.5 mL of 0.1% crystal violet (diluted in 20% ethanol). Aspirate crystal violet and count the number of plaques after the wells are dry. For Supplemental Fig. 5, OT1 and T cells were isolated using the Mojosort CD8 T cell isolation kit (Biolegend Cat. No. 480008). After CD8 negative selection, the cells were labeled with VPD or CFSE to assess proliferation. 5e5-1e6 isolated cells were transferred into immunized mice 3 days before harvest. For OT1 or gBT divisions were (percent divided) was calculated as previously described [77] using the equation fraction diluted =∑i1Ni2i/∑i0Ni2i=∑1iNi2i/∑0iNi2i, where *i* is the generation number (0 is the undivided population), and N_i is the number of events in generation *i*.

Supplementary Files

This is a list of supplementary files associated with this preprint. Click to download.

- Supplementarymaterials81823.docx
